# Expression and regulation of the CXCL9-11 chemokines and CXCR3 receptor in Atlantic salmon *(Salmo salar)*

**DOI:** 10.3389/fimmu.2024.1455457

**Published:** 2024-09-05

**Authors:** Natalia Valdés, Daniela Espinoza, Claudia Pareja-Barrueto, Nicole Olate, Felipe Barraza-Rojas, Almendra Benavides-Larenas, Marcos Cortés, Mónica Imarai

**Affiliations:** ^1^ Centro de Biotecnología Acuícola, Departamento de Biología, Facultad de Química y Biología, Universidad de Santiago de Chile, Santiago, Chile; ^2^ Departamento de Hematología y Oncología, Pontificia Universidad Católica de Chile, Santiago, Chile

**Keywords:** chemokine, teleost, CXCR3, CXCL9, CXCL10, CXCL11, *Salmo salar*, fish immunity

## Abstract

Chemokines are cytokines that mediate leukocyte traffic between the lymphoid organs, the bloodstream, and the site of tissue damage, which is essential for an efficient immune response. In particular, the gamma interferon (IFN- γ) inducible chemokines CXCL9, CXCL10, and CXCL11, and their receptor CXCR3, are involved in T cell and macrophage recruitment to the site of infection. The nature and function of these chemokines and their receptor are well-known in mammals, but further research is needed to achieve a similar level of understanding in fish immunity. Thus, in this study, we seek to identify the genes encoding the components of the Atlantic salmon (*Salmo salar*) CXCL9, CXCL10, CXCL11/CXCR3 axis (CXCL9-11/CXCR3), predict the protein structure from the amino acid sequence, and explore the regulation of gene expression as well as the response of these chemokines and their receptor to viral infections. The *cxcl9*, *cxcl10*, *cxcl11*, and *cxcr3* gene sequences were retrieved from the databases, and the phylogenetic analysis was conducted to determine the evolutionary relationships. The study revealed an interesting pattern of clustering and conservation among fish and mammalian species. The salmon chemokine sequences clustered with orthologs from other fish species, while the mammalian sequences formed separate clades. This indicates a divergent evolution of chemokines between mammals and fish, possibly due to different evolutionary pressures. While the structural analysis of the chemokines and the CXCR3 receptor showed the conservation of critical motifs and domains, suggesting preserved functions and stability throughout evolution. Regarding the regulation of gene expression, some components of the CXCL9-11/CXCR3 axis are induced by recombinant gamma interferon (rIFN-γ) and by Infectious pancreatic necrosis virus (IPNV) infection in Atlantic salmon cells. Further studies are needed to explore the role of Atlantic salmon CXCL9-11 chemokines in regulating immune cell migration and endothelial activation, as seen in mammals. To the best of our knowledge, there have been no functional studies of chemokines to understand these effects in Atlantic salmon.

## 1 Introduction

Chemokines are chemotactic cytokines that regulate migratory patterns and the localization of immune cells in tissues and organs (1). They constitute the most prominent family of cytokines, consisting of approximately 50 chemokine ligands in humans and mice (2). Chemokines are small proteins (8 to 12 kDa) with highly conserved domains. They have a conserved secondary and tertiary structure due to their highly conserved cysteine residues pairing up to form disulfide bridges crucial to maintaining structural integrity and chemokine binding to their cognate receptor (3). At the amino acid sequence level, there is a high divergence with identities ranging between 20% to 90% (1, 4). Although the nature and function of chemokines and their receptors are well-known in mammals, further research is needed to achieve a similar level of understanding in fish immunity.

The first chemokine gene identified in teleost fishes was *ck1*, described in rainbow trout (*Oncorhynchus mykiss*) in 1998 (5). Since then, the identification of fish chemokine orthologs and the characterization of their role have been more complex than expected, mostly because of the whole genome duplication processes occurring in fish and because chemokines evolve faster than other immune genes (6). Accordingly, many more chemokine sequences have been identified in fish than in mammals (6), and definitive homologies have been established only for those chemokines with well-conserved roles (7). Among the most studied chemokines are those of the CXC subfamily with the chemotaxis function for monocytes and lymphocytes. Twenty-five CXC chemokines have been identified in zebrafish (*Danio rerio*), and more than ten in catfish (*Ictalurus punctatus*), rainbow trout, Atlantic salmon, and yellow croaker (*Larimichthys crocea*) (7, 8). With the availability of genome sequences for fish species, phylogenetic analyses have revealed a greater number of genes and complexity in the CXCL9-11 chemokine family. Studies have identified six clades in teleost CXC chemokine sequences: CXCa, CXCb, CXCc, CXCd, CXCL12, and CXCL14. Most fish chemokines form unique branches distinct from human chemokines, suggesting they emerged after fish and mammals diverged. Notably, only CXCL12 and CXCL14 have clear orthologs between fish and humans (8–11). However, chemokines for each clade have not been identified in all species. For example, in rainbow trout, only members of CXCa, CXCb, and CXCd have been reported (11).

The CXCL9-11 chemokines and the CXCR3 receptor have essential roles in Th1-type immune response, migration and activation of CD8 T cells and macrophages. Therefore, they are involved in the clearance of intracellular pathogens (2). Genes encoding the CXCL9-11 chemokines and the CXCR3 receptor have been identified in several fish species [revised in Valdés et al., 2022 (12)]. Translated sequences showed that CXCL9, CXCL10, and CXCL11 contain two conserved cysteine residues separated by one random residue in their N-terminal sequence with no-ELR (glutamic acid, leucine, arginine) chemokine motif present (7, 13)*. Cxcl10* was reported to be transcriptional expressed in lymphoid and non-lymphoid tissues of the rainbow trout (Laing et al., 2002) and its expression showed upregulation in response to IFN-γ (14) and by polyinosinic-polycytidylic acid (poly I: C) but not by lipopolysaccharides (LPS) (15), suggesting a role in viral defense (16, 17). In addition, *cxcl10* was not induced by the Infectious pancreatic necrosis virus (IPNV) but by the viral hemorrhagic septicemia virus (VHSV), indicating a pathogen-specific regulation (16). The expression of *cxcl11* has also been reported in rainbow trout after infection with *Yersinia ruckeri* and *Ichthyophthirius multifiliis* (18, 19). There are few studies on Atlantic salmon. Among what is known, transcriptomic analyses showed that CXCL9 had low expression levels following amoebic gill disease compared to uninfected animals (20). Additionally, the CXCL10 chemokine has been reported in cell lines derived from Atlantic salmon head kidney leukocytes (SHK-1 and TO) (21). In other salmonid fishes, such as Brown trout (*Salmo trutta*), there are few studies of CXCL9, -10, and -11 chemokines (22, 23).

*Cxcr3* has been also characterized in important fish species. For example, in grass carp (*Ctenopharyngodon Idella*) (24), in zebrafish (25) and rainbow trout (26). There are two genes, *cxcr3.1* and *cxcr3.2*, encoding CXCR3 in rainbow trout. Both genes are expressed in various tissues, but *cxcr3.1* is expressed at a significantly higher level than *cxcr3.2* in the thymus, adipose fin, caudal kidney, cephalic kidney, gonad, and spleen (27). On the other hand, *cxcr3.2* is expressed at higher level than *cxcr3.1* in caudal fins, liver, and blood (27). Regarding expression regulation, *cxcr3.1* is up-regulated by poly I:C, IL-1beta, and TNF-alpha, while the expression of *cxcr3.2* is negatively regulated by poly I:C and peptidoglycan (PGN) (27, 28). CXCR3 transcript has been observed in macrophages, contributing to macrophage polarization in ayu (*Plecoglossus altivelis*), grass carp (*Ctenopharyngodon idella*) and spotted green pufferfish (*Tetraodon nigroviridis*) (29).

In this study, our aim was to examine the CXCL9-11/CXCR3 axis of Atlantic salmon, which is a species of fish with high commercial value and serves as a model for salmonid studies. Our research involves identifying the genes encoding the CXCL9-11/CXCR3 axis, predicting the protein structure from the amino acid sequence, and exploring the regulation of gene expression as well as the response of these chemokines and their receptor to viral infections.

## 2 Materials and methods

### 2.1 Phylogenetic analysis

The homologous sequence was searched for each of the amino acid sequences of interest for each target using the BLAST Version 2.7.1 software (30). The sequences best aligned with the gene target sequences were downloaded, and the repeated sequences were removed. The data sets used contained 94 sequences for the analysis of the ligands (CXCL9, CXCL10 and CXCL11) and 64 sequences for the receptor (CXCR3). Sequences were aligned with MAFFT software version 7.409 software (31). The alignment was then visualized with the Geneious Prime Version 2019.0.4 software (32) to see if there were very short and misaligned sequences. Uninformative areas were removed by eliminating all positions with gaps using Mega Version 6.06 software (33). Finally, the tree was built with the FastTree Version 2.1 software using the maximum likelihood method (34) and the Jukes-Cantor model of nucleotide evolution.

### 2.2 Protein modeling

Protein structures of Atlantic salmon CXCL9-11 and CXCR3 were modeled using SWISSMODEL (35), PEPFOLD (36), and MODELLER v9.13 (37). The whole sequence was modeled for each protein studied using a combination of bioinformatics tools. SWISSMODEL was used for initial template-based modeling. Due to the structures of these proteins are unavailable, comparative modeling of CXCL9 (PDB ID 1PLF), CXCL10 (PDB ID 1O7Y), CXCL11 (PDB ID 1QNK), and CXCR3 (PDB ID 3ODU) structures was built using as target Atlantic salmon sequences NCBI XP_014009849.1, NCBI XP_013983422.1, NCBI XP_013998930.1, and NCBI NP_001133965.1, respectively. PEPFOLD, using an ab initio algorithm, was used for all the peptide sequences without 3D structure in the amino and carboxyl terminus. MODELLER was used to build the complete model using information from multiple templates by the above tools (comparative and ab initio modeling), mainly a 3D structure from comparative modeling and two shorter 3D structures from the ab initio method. Twenty models were built for each protein model using MODELLER. The best model was selected according to the lowest energy using the YASARA2 force field (38) and Molprobity score (39) for several stereochemical quality parameters. The final models had the best quality according to the Molprobity score and a minimum of 97.8% amino acid residues in the Ramachandran plot’s favored and additional allowed regions.

### 2.3 Molecular system preparation and molecular docking

All molecular docking was performed using HADDOCK (40). The residues involved in protein-ligand binding were defined from the literature. In the receptor-binding domain, the following residues were involved: 36, 37, 38, 39, 40, 41, 42, 43, and 44. Meanwhile, residues 12, 13, 14, 15, 16, and 17 were involved in the chemokine-binding (41). Additionally, non-restrictive docking was performed, and the lowest energy dockings matched the interactions with the selected residues. On the other hand, the following were the residues suggested as restricted: 70-80, 102-115, 138- 157, 176-212, 233-252, 275-300, these residues correspond to the receptor regions within the cell membrane, so binding in that area was restricted.

### 2.4 Culture conditions for CHSE-214 and SHK-1 cells

The Chinook salmon (*Oncorhynchus tshawytscha*) embryo-derived cell line CHSE-214 (ATCC CRL-1681) was grown at 18°C in minimal essential medium (MEM) supplemented with 10% fetal bovine serum (FBS) (Hyclone), 1 mM Hepes (Corning), 0,1 mM non-essential amino acids (Corning) and 50 μg/mL gentamicin (USBiological). The SHK-1 cell line (ECACC 97111106) (42) from the head kidney of Atlantic salmon, described as macrophage-like cells, was grown at 18°C in Leibovitz’s 15 medium supplemented with 10% FBS, 4 mM L-glutamine and 40 μM of 2-mercaptoethanol. Cells were grown in culture bottles (SPL) and propagated by washing twice with PBS and adding TrypLE Express solution (Gibco) to detach the cells.

### 2.5 Recombinant protein production IFN-γ

Competent Escherichia coli BL21 Star (DE3) cells (Invitrogen/LifeTechnologies) were transformed with the pET-15b-ssIFN-γ1 vector (Ictio Biotechnology). Transformed bacteria were grown in LB Broth liquid medium (MO BIO) and 0.1 mg/mL of ampicillin at 37°C under agitation (180 rpm) until an OD 600 nm of 0.6 was reached. The bacterial culture was induced by adding 1 µM isopropyl 1-thio-b-D-galactopyranoside (Bioline) for 3 h at 30°C, added during the exponential growth of the bacteria. Bacteria were precipitated by centrifugation at 6,000 *g*, 4°C, 40 min, and the pellet was resuspended in a solubilization buffer containing 20 mM Tris-HCl pH 8.0, 0.5 M NaCl, 6 M guanidinium chloride, and EDTA-free protease inhibitors (Roche). The bacteria were disrupted using an ultrasonic homogenizer called Omni Sonic Ruptor (OMNI International) at 4°C with 10 pulses of 20 seconds and 12 watts. After centrifugation at 6000 g for an hour at 4°C, the soluble fraction was recovered, and the recombinant protein was purified using Fast Protein Liquid Chromatography (FPLC) (AKTApurifier). The process involved loading the soluble protein fraction onto a 1 mL nickel column Histrap FF crude (Cytiva) and washing it with 5 column volumes of binding buffer (20 mM Tris-HCl pH 8.0, 0.5 M NaCl, 6 M guanidinium chloride). The bound protein was eluted using a 15-column volume linear gradient of 20--500 mM imidazole, and the fractions were detected at 280 nm. The fractions containing the recombinant protein were collected and pooled. The buffer was exchanged using 10 kDa cutoff Amicon Centrifugal Filter Units with phosphate-buffered saline (PBS), and the protein solution was concentrated, quantified, aliquoted, and stored with 20% glycerol at -40°C until use. After protein purification, samples were analyzed by denaturing SDS-PAGE, and then stained with Coomassie Brilliant Blue (43). The size of the purified protein was between 17 and 20 kDa (**Supplementary Figure 1**).

### 2.6 RNA extraction and cDNA synthesis

The organs (50 mg) or cell pellets were resuspended in 1 mL of TRIzol^®^ Reagent (Ambion^®^, Life Technologies). The organs were homogenized using a tissue cell disruptor (Omni International) while the cells were homogenized by passing them through the pipette tip multiple times. To extract the total RNA, we followed the manufacturer’s protocol. The extracted RNA was then resuspended in diethyl pyrocarbonate–treated water (Invitrogen) and quantified. We treated RNA samples (2 μg) with DNase I (AMPD1-1 KT, Sigma) and synthesized cDNA using reverse transcriptase Moloney murine leukemia virus (Sigma), oligo (dT) (Promega), and dNTPs (Promega) in accordance with the manufacturer’s instructions. We kept the RNA samples at −80°C and cDNA at −20°C until use (44).

### 2.7 Quantitative PCR

The real-time PCR reactions were performed in 96-well plates (Axygen) covered with optical caps (Axygen) in a AriaMx Mx3000P (Stratagene). PCR reaction efficiencies were determined by generating cDNA standard curves using serial dilutions (1:10) of a mix of total cDNA synthesized from RNA total isolated from head kidney of Atlantic salmon (45). **Table 1** shows the target gene, primer sequences, the calculated efficiencies and accession number. The expression of three different reference candidate genes (*ef1α, 18s, and β-actin*) was tested for stability using the BestKeeper software (46). Thus, the *β-actin* was chosen for further analyses because of their lower variation among all the samples. The primer efficiency was determined according to Pfaffl (45), and the presence of a PCR product was verified in the melting curve for each set of primers. Each reaction was carried out in 10 μL final volume containing 5 μL SsoAdvanced Universal SYBR Green Supermix (BIO RAD), 0.5 uL forward primers (10 uM), 0.5 uL reverse primers (10 uM), 3 μL ultrapure distilled water (Invitrogen), and 1 μL cDNA (diluted 1:10 for housekeeping gene). The cycling conditions were 95°C for 10 min, followed by 40 cycles of 95°C for 15 s, 58–62°C for 15–30 s, and 72°C for 30 s (depending on the primer set). Data were analyzed using AriaMX quantitative PCR software (Agilent Technologies). The normalized relative expression (NRE) of the different genes was calculated using the Pfaffl equation, which accounts for primer efficiency (E) with the formula (E^-ΔΔCT^) (45) method normalizing the expression levels of target genes against the *β-Actin*. The mathematical representation of the NRE equation is:


$$NRE=\frac{{\left(E_{target}\right)}^{\Delta CT\;target\left(control-treated\right)}}{{\left(E_{ref}\right)}^{\Delta CT\;ref\left(control-treated\right)}}$$

**Table 1:** Chemokine axis CXCL9-11/CXCR3 primers.

Target gene	Sequence (5’ – 3’)	Tm (°C)	Efficiency in kidney	Accession number
*cxcl9*	F: *CTCTGTGGTCACCCTAAGGC* R: *CTGCGTGGAAGAAAAC*	57.4 57.2	110	XR_006760298
*cxcl10*	F: *AGGAGTGTGCAGTAAATCTGTGAAC* R: *CTCATGGTGCTCTCTGTTCCA*	57 56.8	109	EF619047.1
*cxcl*11	F: *AGAGGCTCCATTTGCCAAGA* R: *GGCTGTCTTCAGGCAGTTTT*	56.7 55.8	91	XM_014143446.2
*cxcr3*	F: *TAGAAACTTCCGGCGACACG* R: *TTGGGTTCAACGTCCCCTTC*	57.1 57.6	110	XM_045718706.1

### 2.8 Fish and organ dissection

Atlantic salmon (*Salmo salar*) were obtained from Chilean farms. The fish were maintained in tanks with a freshwater system at a biomass of 12 kg/m^3^, at 10–12°C with continuous aeration and fed with commercial pellets twice a day. For extraction of organs and tissue, fish were euthanized with an overdose of tricaine methanesulfonate 80% (Dolical 80%, Centrovet). The organs were snap-frozen in liquid nitrogen and stored at − 80 °C for further analyses.

### 2.9 RT-qPCR analysis from salmon tissues

To examine the levels of CXCL9-11/CXCR3 transcripts in Atlantic salmon tissues, the head, middle and distal kidney, gut, gill, muscle, spleen, liver, brain, and heart were dissected from 4 fish (47). RNA preparation and cDNA synthesis were performed as described before (44). Real-time PCR analysis was performed in 96-well plates (Axygen) using a AriaMX quantitative PCR software (Agilent Technologies). Each reaction was carried out in 10 μL final volume containing 5 μL of SsoAdvanced Universal SYBR Green Supermix (BIO RAD), 0.5 uL forward primers (10 uM), 0.5 uL reverse primers (10 uM), 3 uL of ultrapure distilled water (Invitrogen), and 1 uL of cDNA. No template controls were done for all real-time PCR reactions to examine potential contaminations. All used primers showed an amplification efficiency between 90% and 110%. The primer sequences are listed in **Table 1**. The expression level was normalized to that of *β-actin* and expressed as relative to that of lowest level (liver).

### 2.10 Stimulation of SHK-1 cells with rIFN-γ

To stimulate SHK-1 (42) cells with rIFN-γ, 4 x 10^5^ SHK-1 cells were seeded in each well of 6-well plates and cultured in a supplemented Leibovitz medium (L-15, Sigma-Aldrich) with 10% FBS (Hyclone), 40 μM β-mercaptoethanol, 50 μg/mL gentamicin for 24 hours at 18°C. rIFN-γ was added at a concentration of 50 ng/mL and incubated for 3, 6, 9, and 12 hours. A control of unstimulated cells (without rIFN-γ) was also included. Total RNA was extracted after the treatment to quantify the expression of genes using the RT-qPCR method. The test was carried out with three biological replicates, each with three technical replicates.

### 2.11 Treatment of SHK-1 cells with poly I:C

SHK-1 cells were grown to 80% confluence in 6-well plates at 18°C. Then, Lipofectamine 2000 reagent (Thermo Fisher) was used to transfect the cells with 10 μg/mL of poly I:C of low molecular weight, ranging from 0.2 kb to 1 kb (InvivoGen), following the manufacturer’s instructions. Untreaed cells were used as control. Total RNA was extracted 24 hours after the treatment to quantify the expression of the genes by the RT-qPCR method. Each condition was evaluated in triplicate.

### 2.12 Propagation and titration of the IPN virus

The Infectious Pancreatic Necrosis Virus (IPNV) was grown in monolayers of CHSE-214 cells (48). To infect the cells, an IPNV inoculum was added and left for an hour for adsorption. After 1 hour, the inoculum was removed with the aid of a micropipette, the medium and the virus that did not enter the cells were removed. The cells were then maintained in MEM medium with 2% FBS. The cell were then incubated at 18°C until a cytopathic effect was observed, which usually took around 48 to 72 hours after infection. The viral titer present in the supernatant of the infected cells was determined using the lysis plate method (49).

### 2.13 Infection of SHK-1 cells with IPNV

SHK-1 cells were grown to 80% confluence in 6-well plates in L-15 medium supplemented (FBS 10%, b-mercaptoethanol 40 μM, gentamicin 50 μg/mL) and incubated overnight at 18°C. Cells were infected with IPNV using an MOI of 0.1 plaque-forming units per cell (PFU/cell). The cells were collected 24, 48, and 72 hours after infection to quantify gene expression by the RT-qPCR method. Experiments were performed in triplicate.

### 2.14 Statistical analysis

GraphPad Prism 9.0.2 for MacOSX was used for statistical procedures and graph drawing. Statistical gene expression analyses were performed using the Mann-Whitney U test, one way ANOVA or t-test with Welch’s correction. p<0.05 was considered statistically significant.

## 3 Results

### 3.1 Phylogenetic analysis of the chemokines of the CXCL9-11/CXCR3 axis of Atlantic salmon

**Figure 1:**
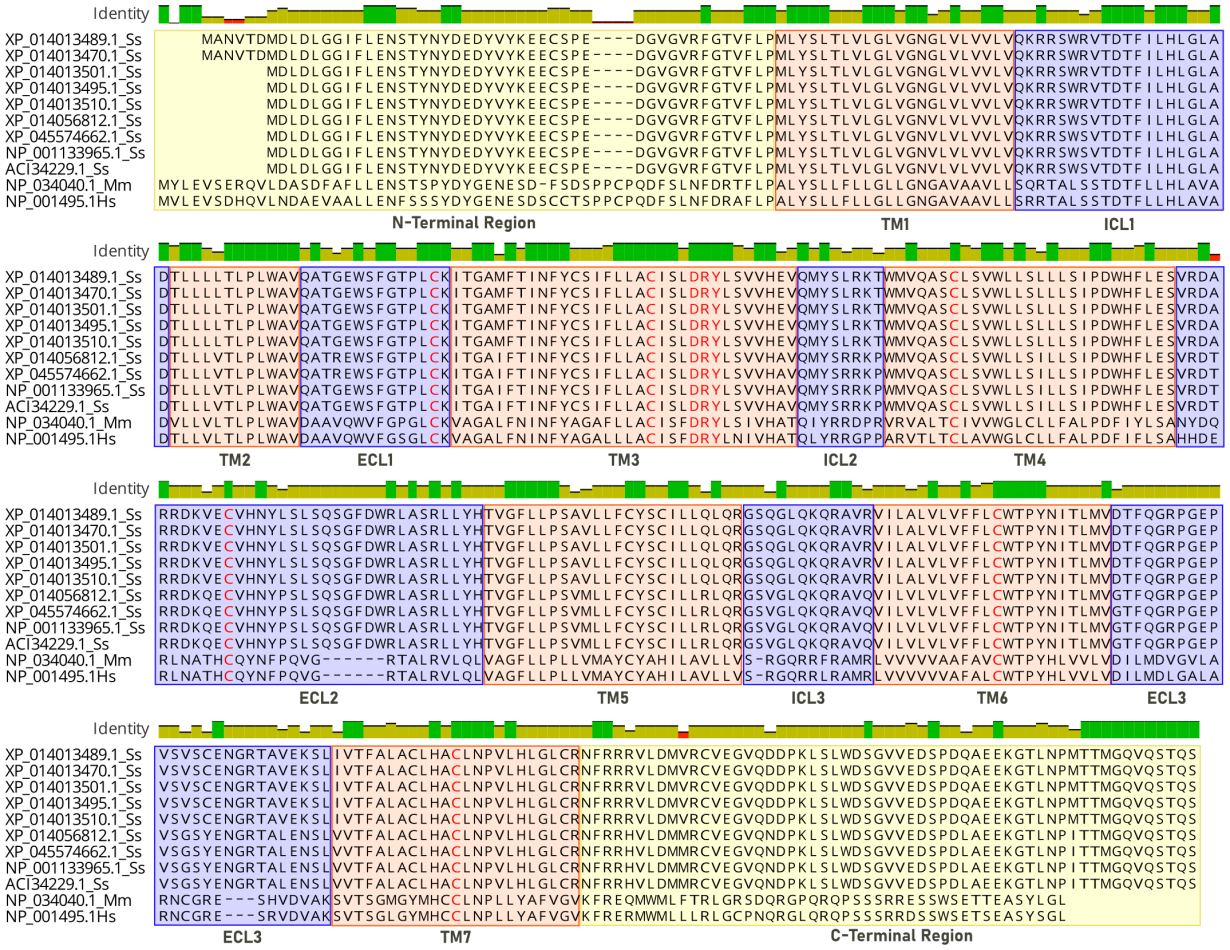
Sequence alignment of CXCR3 receptors. Analysis is based on the structure of CXCR3 chemokine receptors, where sequences 1-9 correspond to the Atlantic salmon (Ss) receptor, sequence 10 corresponds to mouse (Mm), and sequence 11 to human (Hs). Transmembrane (TM) intra (ICL) and extracellular (ECL) domains are colored in orange and blue, and conserved motifs colored in red.

The amino acid sequences translated from the predicted sequences of the chemokines CXCL9 (XP_014009849.1), CXCL10 (XP_013983422.1), and CXCL11 (XP_045551194.1) from Atlantic salmon were submitted to phylogenetic analysis. The analysis shows that the CXCL9-11 chemokine genes from salmon clustered with their respective orthologs from other fish species (**Figure 1A**). The amino acid sequence of Atlantic salmon CXCL9 shares a clade with the ortholog of *S. trutta* (XM_029753354.1); additionally, in the same clade, there are different species of *Salvelinus* (*alpinus* and *namaynchus*) and 2 species of fish from the genus *Oncorhynchus* (*nerka* and *gorbuscha*), both of which belong to the salmonid family, like Atlantic salmon. The sequence of CXCL10 chemokine (XP_013983422.1) groups in the same clade with *P. altivelis*, *A. alosa*, *C. magur*, *S. grahami*, and *C. carpio*. As for the CXCL11 chemokine, it is grouped in the same clade with *S. trutta* (XP_029619627.1). In addition, the analysis included sequences from mammals (*H. sapiens* and *M. musculus*). The sequences from both organisms cluster together for the chemokine CXCL9 in a clade that is not connected to the CXCL9 sequences from fish, while the sequences of CXCL10 and CXCL11 from both organisms share a clade that is clustered with the sequences of CXCL10 chemokines from fish. In the phylogenetic analysis of the CXCR3 receptor CXCR3 (**Figure 1B**), the amino acid sequences used was NP_001133965.1. This sequence is grouped in the same clade with a sequence of the CXCR3 receptor from *Salmo trutta* (XP_029600722.1). In this branch, organisms mainly belong to the Salmonidae family, specifically the genera Salmo, Oncorhynchus, and Salvelinus. The mammal sequences of mouse (*M. musculus*) and human (*H. sapiens*) were included in the analysis. Additionally, other chemokine receptors’ sequences were added, and they mostly grouped together with the CXCR3 receptor sequences. Altogether, in this analysis we have successfully established the orthologous relationships between the predicted sequences of CXCL9-11 axis of salmon and the genes from various fish species with diverse taxonomy.

### 3.2 Protein structure of the CXCR3 receptor of the Atlantic salmon

**Figure 2:**
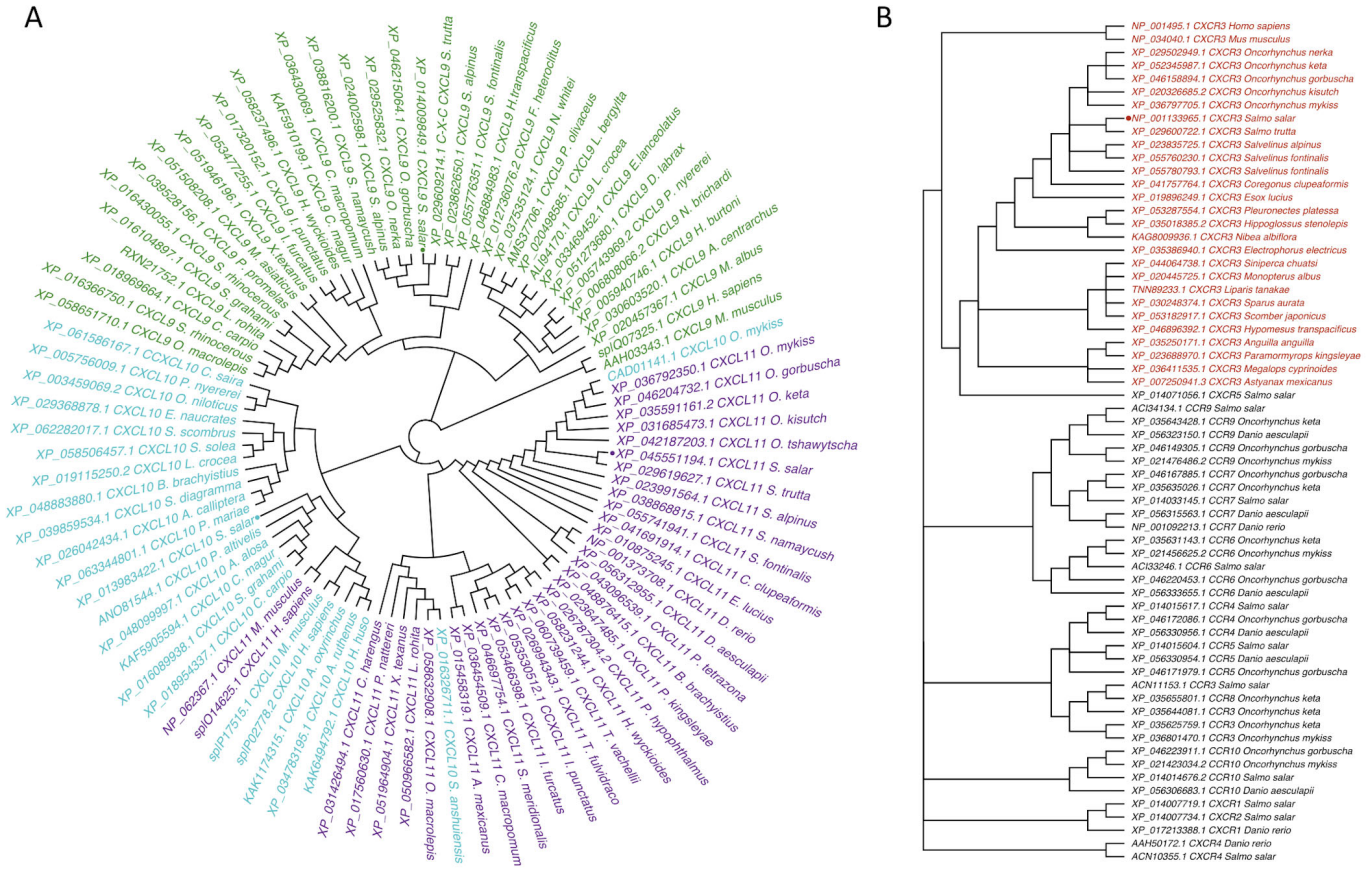
Phylogenetic tree of CXCL9-11/CXCR3 axis genes in Atlantic salmon based on amino acid sequences. **(A)** CXCL9, CXCL10, CXCL11, and **(B)** CXCR3 are highlighted in green, turquoise, and purple, respectively. CXCR is highlighted in red. Sequences from Atlantic salmon (*S. salar*) are indicated with a dot. Amino acid-based phylogenetic analyses were generated using MAFFT Version 7.409 software, and the tree was constructed with the FigTree program using the Bayesian method.

**Figure 3:**
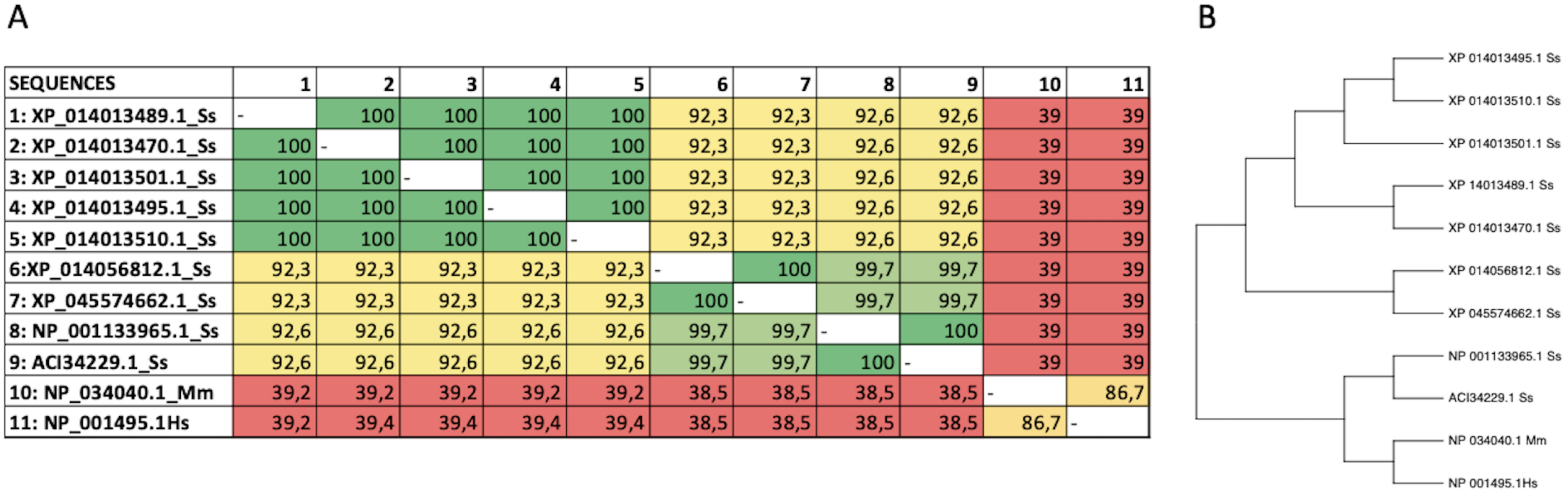
Alignment of protein sequences and phylogenetic tree analysis of CXCR3. **(A)** Multiple sequence alignment of amino acid sequences of CXCR3 from different species. **(B)** Phylogenetic analysis of CXCR3 proteins from different species. The tree was generated using the maximum likelihood method in MEGA 11.0 software, with bootstrap values of 1000 replicates.

In the database, nine protein sequences encoding the CXCR3 receptor of Atlantic salmon were found (ACI34229.1, NP_001133965.1, XP_014056812.1, XP_045574662.1, XP_014013495.1, XP_01401.1, XP _014013510.1, XP_014013470.1 and XP_014 013489.1). A human sequence (Hs NP_001495.1) and a mouse sequence (Mm NP_034040.1) for CXCR3 were also selected for analysis. The Atlantic salmon CXCR3 sequences can be arranged into three groups based on the percentage of identity: one including the sequences_ XP 014013489.1, XP_014013470.1, XP_014013501.1 XP_014013495.1, and XP_014013510. 1 that are equal; other comprising the sequences XP_014056812.1 and XP_045574662.1 that are the same, and the group including the sequences NP_001133965.1 and ACI334229.1 having 100% identity between them. The percentage of identity between the different sequences of Atlantic salmon is greater than 92% (**Figure 2A**). According to the analysis, the three variants identified have amino acid changes that do not affect the receptor’s chemokine binding region. This implies that their presence would not cause any hindrance to the binding of the receptor to chemokines. The human and mouse CXCR3 sequences have 86.7% percentage of identity, while with the Atlantic salmon CXCR3 sequence has approximately 38% identity with the human sequence. This is consistent with the phylogenetic analysis of the receptor sequences, where the Atlantic salmon sequences cluster into three clades (**Figure 2B**). The alignment of sequences was performed to assess the presence of conserved motifs and domains (**Figure 3**). Secondary structure-based analysis and alignment with the murine and human CXCR3 protein sequences showed that the CXCR3 receptor sequences possess seven transmembrane (TM) domains, interspersed by intracellular (ICL) and extracellular (ECL) regions (**Figure 3**). Furthermore, the DRY motif (comprising of aspartate, arginine, and tyrosine), which is essential for signal transduction are also conserved in all variants, as well as the cysteines that allow the formation of intramolecular disulfide bridges key for the folding, stability, and function of the receptor.

**Figure 4:**
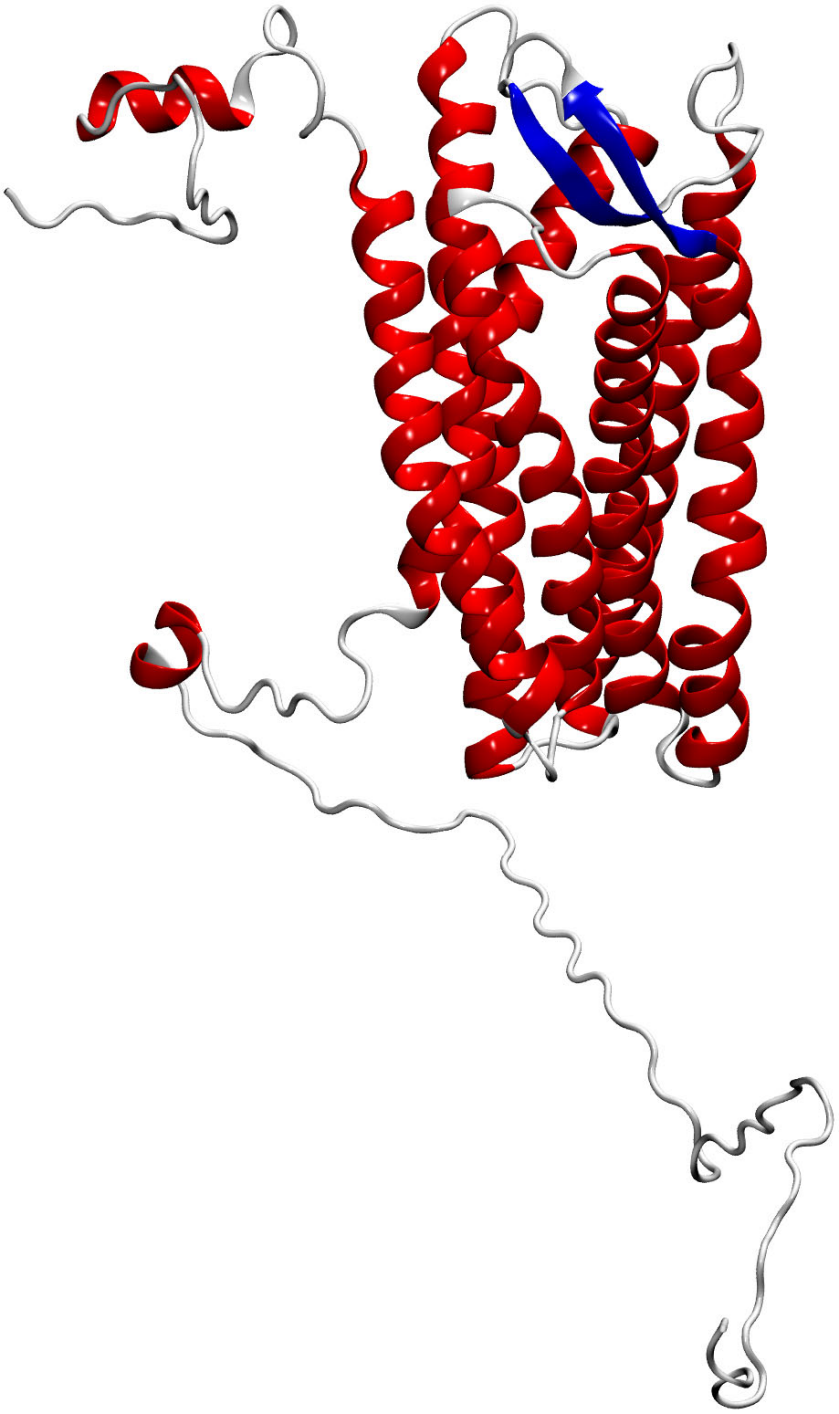
Molecular modeling of Atlantic salmon CXC3 receptor. The CXCR3 chemokine receptor is represented with alpha helices in red, beta sheets in blue, and loops in grey.

CXCR3 was modeled comparatively, using the crystal structure of human CXCR4 with PDB ID 3ODU as a template (**Table 2**). This crystal was chosen because crystal of the CXCR3 were not available, and it has the highest similarity to the amino acid sequence of CXCR3 from Atlantic salmon (NCBI NP_001133965.1). The identity between the Atlantic salmon sequence and the selected crystal structure was 39%, while the similarity based on physicochemical properties was 61%. The protein model of CXCR3 is presented in **Figure 4**. The best model had a Prosa Z-score of −2.77 and 96.7% of residues in the most favored regions. These values suggest that our model had a good energetic and stereochemical quality as well as in the case of CXCL models. The CXCR3 model features seven transmembrane domains and three extracellular loops. A structural superimposition was performed to compare the models of CXCR3 in Atlantic salmon and CXCR4 in humans. The calculations indicated excellent performance, with an RMSD value of 0.60 Å. Thus, we succeeded in generating a high-quality model that is highly similar to the crystal structure of the template.

**Table 2:** Comparative analysis of the CXCL9-11/CXCR3 axis of Atlantic salmon and structures.

Target Proteins	Identity Structure	Similarity Structure	Structure Organism	Accession ID (PDB)
CXCL9	30%	57%	*Bos taurus*	1PLF
CXCL10	47%	61%	*Homo sapiens*	1O7Y
CXCL11	48%	62%	*Homo sapiens*	1QNK
CXCR3	39%	61%	*Homo sapiens*	3ODU

### 3.3 The Atlantic salmon CXCL9, CXCL10 and CXCL11 protein structure defined by *in silico* analysis

**Figure 5:**
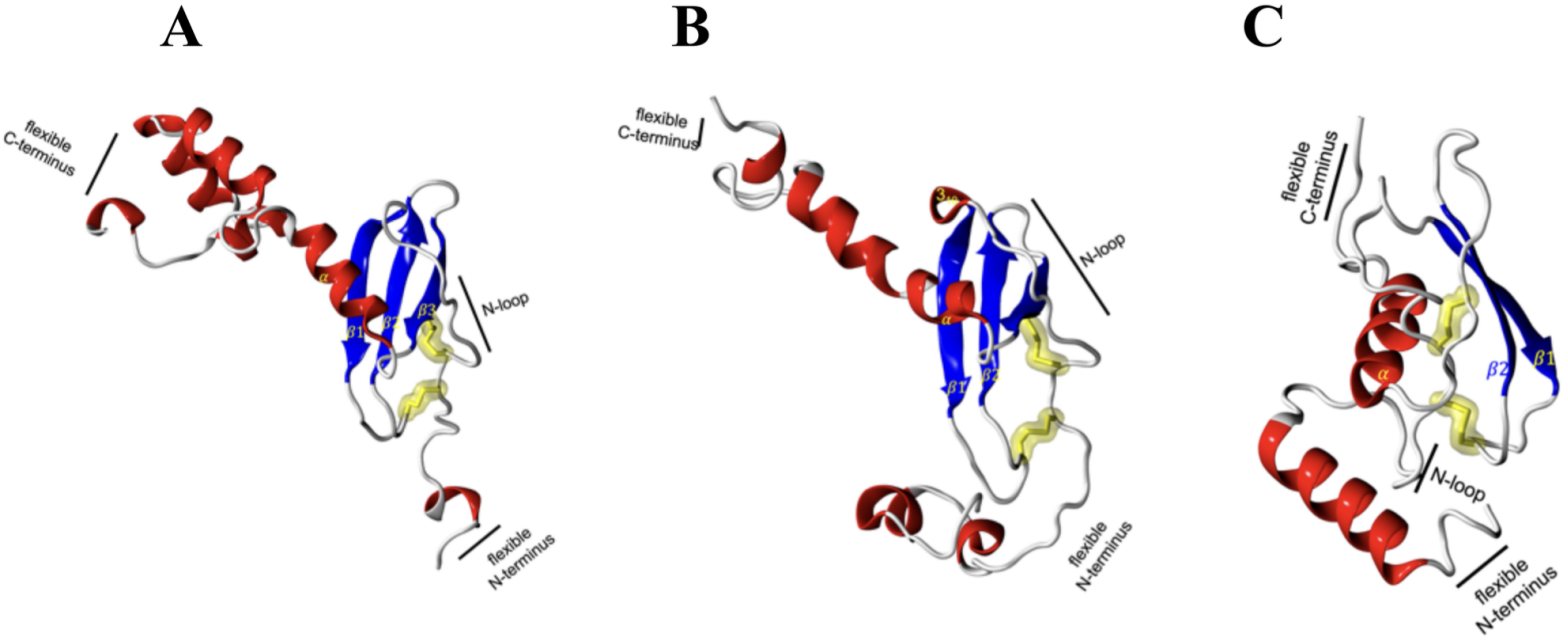
Molecular modeling of Atlantic salmon CXCL9, CXCL10, and CXCL11 chemokines. **(A)** CXCL9, **(B)** CXCL10, and **(C)** CXCL11 chemokines are represented with alpha helices in red, beta sheets in blue, loops in white and yellow highlighting the cysteine residues forming disulfide bonds characteristic of the CXC family of chemokines. The CXCR3 chemokine receptor is represented with alpha helices in red, beta sheets in blue, and loops in white, with highlighted disulfide bond side chains in yellow.

Based on the retrieved amino acid sequences of Atlantic salmon CXCL9, CXCL10, and CXCL11 chemokines, molecular modelling was performed to unveil the conserved 3D-configuration for chemokines. The whole sequence was modeled for each protein studied using a combination of bioinformatics tools as outlined in the Methods. To construct the 3D structures, we modeled the Atlantic salmon sequences NCBI XP_014009849.1, NCBI XP_013983422.1, and NCBI XP_045551193.1, using as templates the crystal structures of CXCL9 (PDB ID 1PLF), CXCL10 (PDB ID 1O7Y), and CXCL11 (PDB ID 1QNK), respectively. As shown in **Table 2**, the chosen crystals were CXCL9 from Bos taurus CXCL10 and CXCL11 from humans. The identity of the Atlantic salmon sequences and the selected crystals was 30.3%, 47%, and 48%, while the similarities based on physicochemical properties were 57%, 61% and 62% for CXCL9, CXCL10, and CXCL11, respectively. The protein models of Atlantic salmon CXCL9, CXCL10, and CXCL11 are shown in **Figure 5**. The cysteines forming two disulfide bonds in the template structures are aligned with the four corresponding cysteines in the CXCL9-11 3D models (highlighted in yellow, **Figure 5**). The best models had Prosa Z-score (50) of -5.01, -1.81, -3.87 and 98, 100, and 90% of residues in most favored regions according to Ramachandran analyses (51). These values suggest the successful generation of high-quality models. CXCL9, -10, and -11 present the classic structure of CXC chemokines characterized by a short N-terminal region, a large core stabilized by two disulfide bonds (yellow), three antiparallel beta-strands (blue), and a C-terminal alpha-helix (red) (**Figure 5**). The core structure is well-ordered, but the N and C-terminals exhibit high conformational flexibility. The models of the three chemokines were compared by structural superimposition. The RMSD values obtained comparing all the model with the respective crystal resulted in 0.771, 0.752 and 1.611 Å, which means a good quality for the comparative modeling. CXCL11 shows a slight difference in third beta-sheet consistent with the RMSD value. The model of CXCL9 with both CXCL10 and CXCL11 resulted in 1.484 and 2.544 Å, respectively, and between CXCL10 and CXCL11 was 2.177 Å which means that all models show high structural similarities. Moreover, visual inspection of the secondary structures evidenced that helices and beta-sheets are well conserved along each model. These assessments suggest that the tertiary structure of the three CXC chemokines is similar, with the main structural differences located at the loop level.

### 3.4 *In silico* analysis of interaction of the chemokines with the CXCR3 receptor of Atlantic salmon

**Figure 6:**
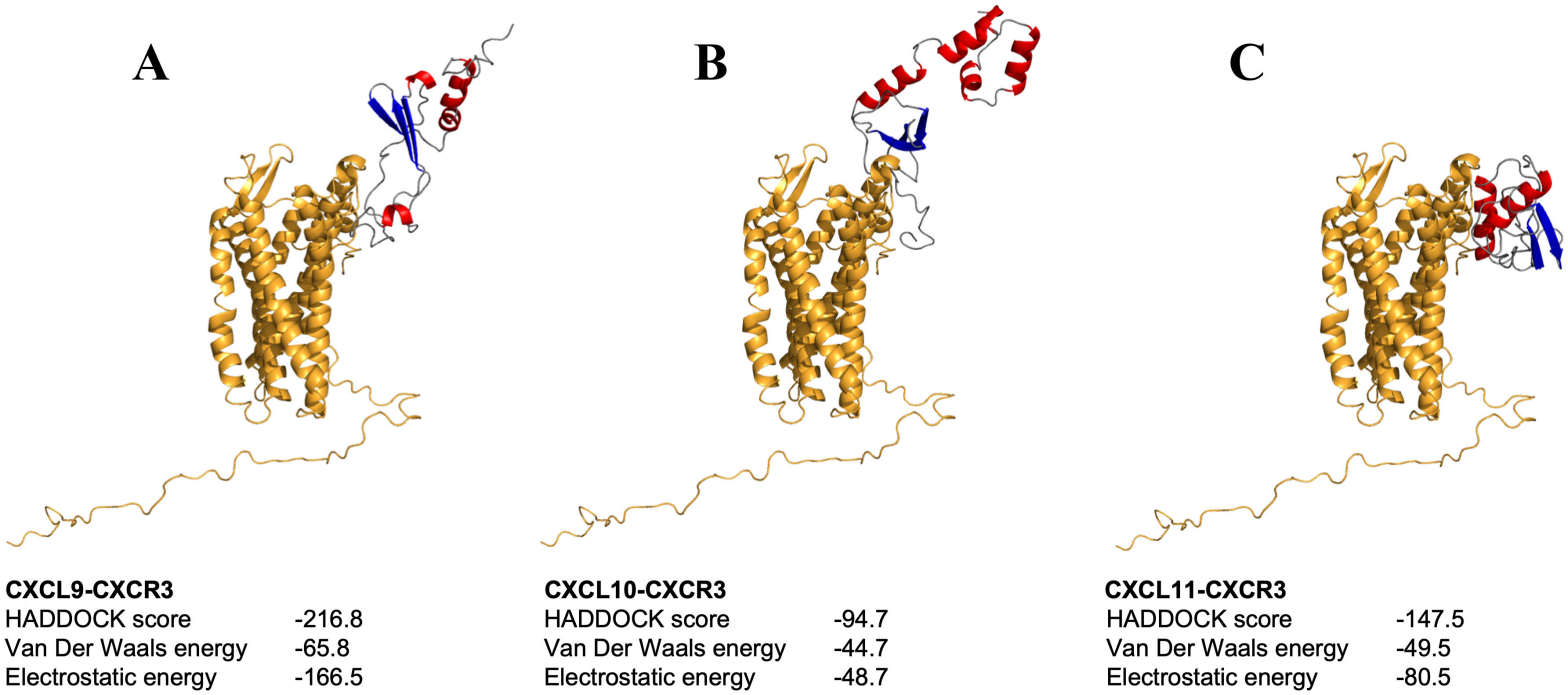
Molecular docking between chemokines-CXCR3 complex. **(A-C)** depict the docking of chemokines CXCL9, CXCL10, and CXCL11 with CXCR3, respectively. Molecules are shown in ribbon representation, with the receptor displayed in orange and chemokines colored by secondary structure.

The analysis of the CXCL9-11/CXCR3 complexes was also performed using the above-mentioned bioinformatics tools. For each complex, the best docking was selected by binding energy according to the HADDOCK score, that is a weighted sum of a variety of energy terms including van der Waals, electrostatic, desolvation, and restraint violation energies (Evdw, Eelec, Edesol, and Eair, respectively) (52). The selected complexes were screened for interacting residues based on hydrophobicity, hydrogen bonding, and salt bridges (**Table 3**). CXCR3 showed the presence of variable interesting outcomes in terms of the conformation and affinity of binding. The best HADDOCK score of CXCL9/CXCR3 interaction was -216.8 Kcal/mol and this complex obtained the best score compared to the other chemokines analyzed. CXCL10-CXCR3 interaction obtained a HADDOCK score of -94.7 Kcal/mol, and -147.5 Kcal/mol for CXCL11-CXCR3. The above differences are given by the type of interactions established, among them *Van Der Waals* and *electrostatics* are the ones that contribute the most to the score. The binding between CXCL9 chemokine and CXCR3 receptor also shows a higher number of interactions, as supported by **Table 3**. As expected, the CXCL9/CXCR3 complex, which has a lower HADDOCK score, has the largest number of interacting residues, forming six hydrophobic interactions, nine hydrogen bonds, and two salt bridges. Our results showed that the N-terminal region of CXCL9 interacts with the N-terminal region of CXCR3 and N-loop 1 of CXCL9 with loop 6 between transmembrane (TM) segment 6 y 7 of CXCR3, CXCL9 loop 2 interacts with CXCR3 N-terminal, CXCL9 loop 3 interacts with CXCR3 loop 7 (**Figure 6A**). The CXCL10/CXCR3 complex shows interactions between the CXCL10 N-terminal and CXCR3 N-terminal and the helix that is part of TM1 (**Figure 6B**). The CXCL11/CXCR3 complex shows interactions between CXCL11 N-terminal with CXCR3 N-terminal and TM1, CXCR3 TM6 interacts with CXCL11 N-loop 1, and CXCR3 loop 6 and TM7 interacts with CXCL11 C-terminal included the terminal helix (**Figure 6C**).

**Table 3:** Main interactions in the chemokine/receptor CXCR3 complexes.

Index	Interaction	Receptor Residue	Chemokine Residue	Distance Å
CXCL9				
1	Hydrophobic	Tyr23	Val22	3.82
2		Tyr23	Phe29	3.87
3		Arg36	Tyr27	3.97
4		Thr290	Asp28	3.47
5		Pro29	Asn60	3.87
6		Tyr285	Glu73	3.97
1	Hydrogen Bond	Tyr23	Phe29	3.33
2		Val25	Cys34	2.72
3		Arg36	Cys36	2.88
4		Phe37	Tyr37	2.70
5		Gly38	Tyr37	2.96
6		Ser282	Arg70	2.70
8		Ser284	Thr71	3.49
9		Ser284	Glu73	3.40
10		Glu286	Glu73	3.37
1	Salt Bridges Ionic	Asp4	Lys24	3.74
2		Glu286	Lys40	5.39
CXCL10				
1	Hydrophobic	Tyr23	Gln20	3.87
2		Ile8	Ala16	3.76
3		Val40	Ala16	3.94
4		Met44	Ala16	3.61
1	Hydrogen Bond	Arg36	Gly15	3.18
2		Val40	Ala16	2.99
3		Gly6	Gln20	2.91
4		Tyr23	Pro22	2.86
CXCL11				
1	Hydrophobic	Phe41	Phe13	3.33
2		Phe37	Leu17	3.53
3		Val35	Leu19	3.87
4		Arg36	Val20	3.87
5		Ile266	Val40	3.75
6		Ala291	Phe84	3.64
1	Hydrogen Bond	Gly6	Thr8	3.81
2		Asn287	Glu23	3.84
3		Ser284	Lys44	2.73
4		Tyr285	Lys91	2.94
5		Ser284	Arg97	2.98

### 3.5 Basal transcription and regulation of *cxcl9, cxcl10*, *cxcl11*, and *cxcr3* expression in Atlantic salmon

**Figure 7:**
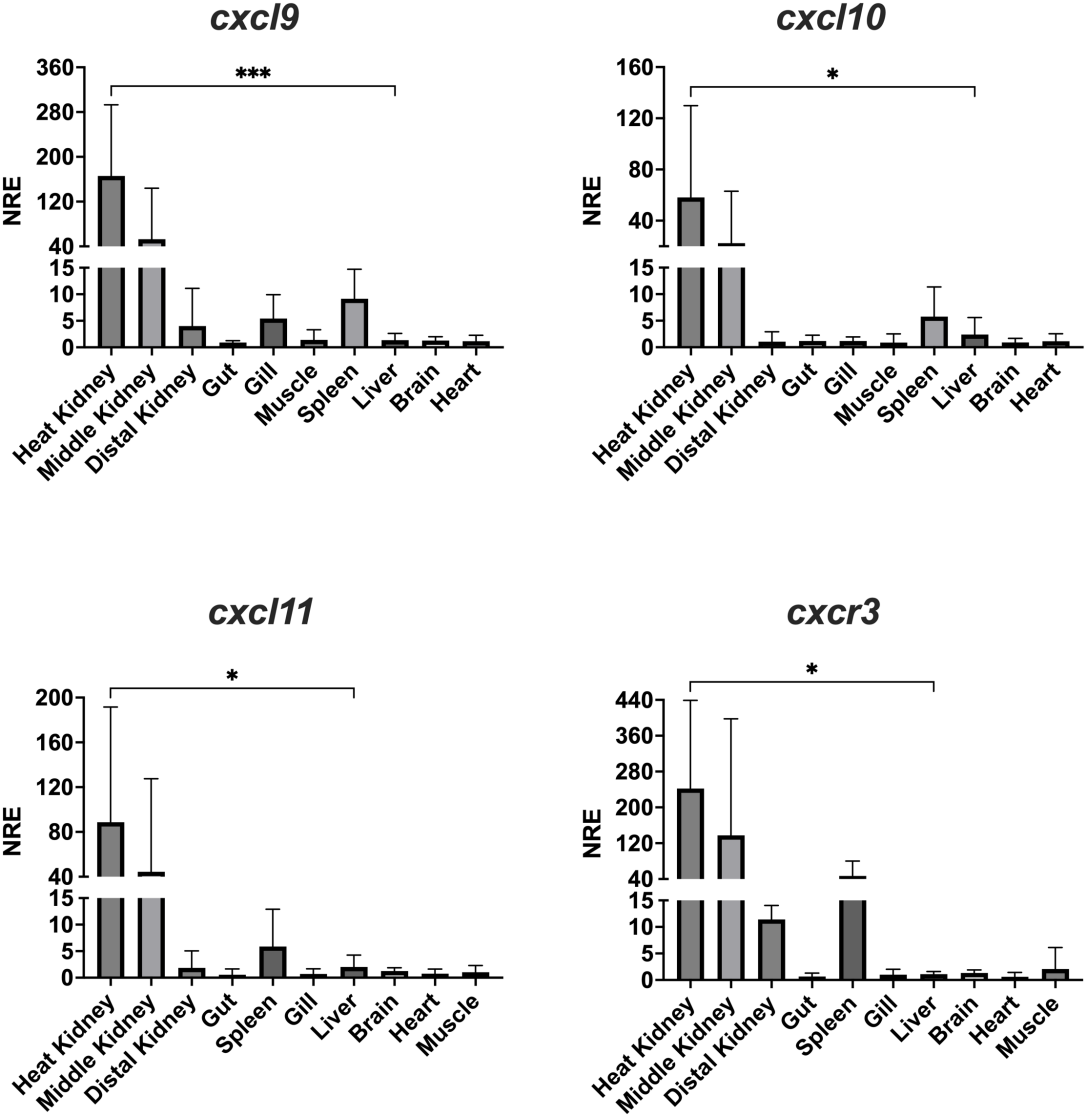
Tissue distribution of transcript expression of CXCL9-11/CXCR3 axis in Atlantic salmon. The expression level of the receptor transcripts was determined by real-time RT-PCR in lymphoid and non-lymphoid tissues obtained from 4 fish. Gene expression data were normalized to *β-Actin*. Data represent the mean ± SD of relative expression levels to expression in the liver. Differences between groups were determined with one way ANOVA followed by Dunnet *post hoc* test. * p < 0.05; *** p < 0.001

The transcriptional expression of the Atlantic salmon *cxcl9, cxcl10, cxcl11* and *cxcr3* genes were examined by RT-qPCR in several tissues obtained from healthy fish. The expression of the four genes was observed in all the analyzed lymphoid tissues of Atlantic salmon, including the head, middle and distal kidney, spleen, gills, and intestine (**Figure 7**). Transcripts of *cxcl9, cxcl10, cxcl11*, and *cxcr3* were found in all tested tissues, but with higher expression levels in the head kidney and middle kidney.

**Figure 8:**
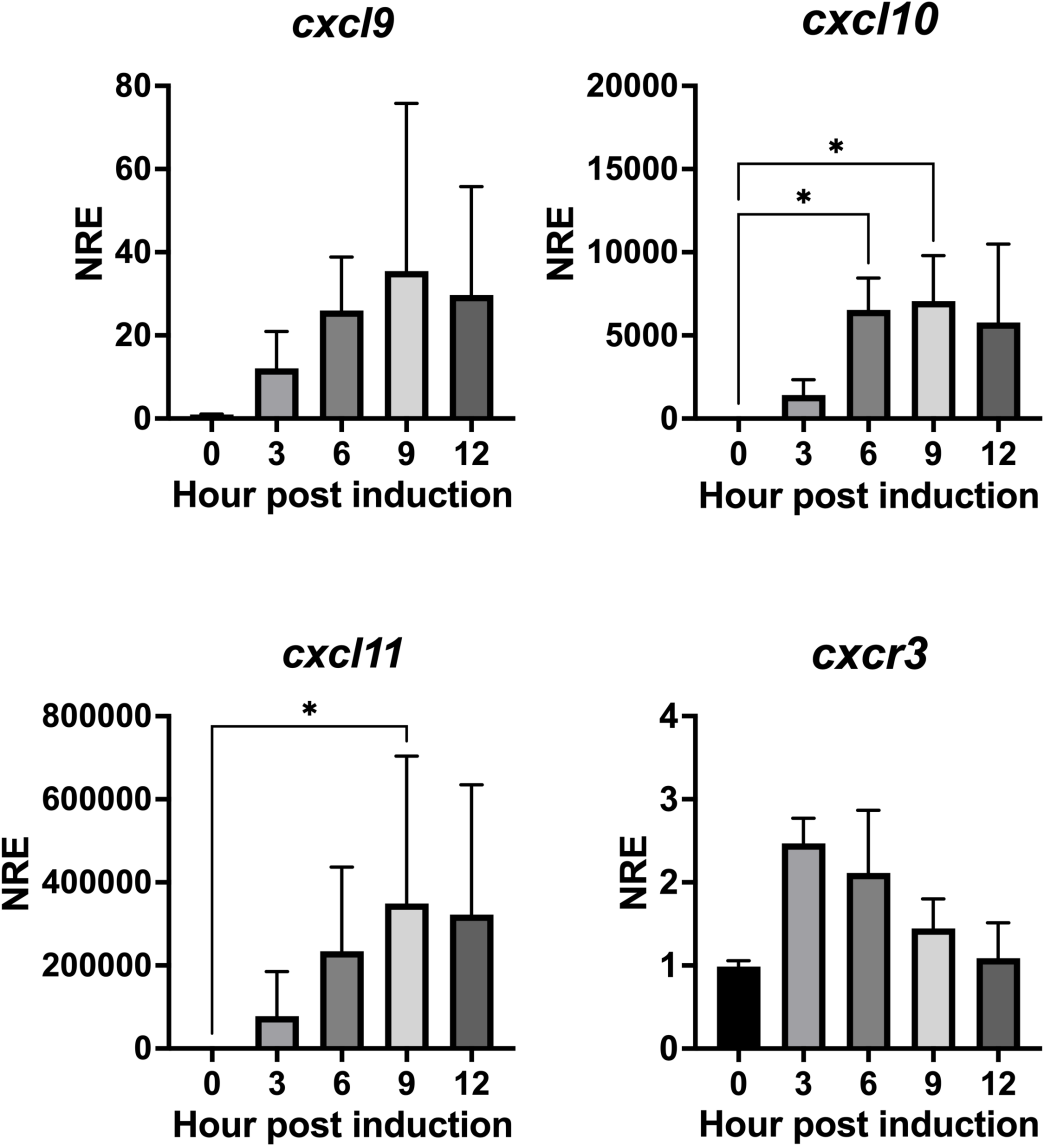
rIFN-γ modulates the expression of genes *cxcl9, cxcl10, cxcl11* and *cxcr3* in SHK-1 cells. Cells were treated with recombinant IFN-γ for 3, 6, 9, and 12 h. Specific mRNA levels were measured by RT-qPCR and expressed gene expression was reported as relative to *β-Actin* expression (reference gene) and normalized with the relative expression of each gene in untreated cells (control group). The values obtained for each condition were expressed as normalized relative expression (NRE) ± standard deviation (SD) of 3 independent experiments (n=3). Differences between groups were determined with Kruskal-Wallis followed by Dunnet *post hoc* test. * < 0.05.

We investigated whether IFN-γ can induce transcriptional expression of the CXCL9-11 axis genes in Atlantic salmon. To accomplish this, we utilized the SHK-1 macrophage-like cell line, which was derived from the kidney of Atlantic salmon, and stimulated the cell line with recombinant IFN-γ at a concentration of 50 ng/mL (53). This particular dose was chosen because it has been demonstrated to produce the most significant effect of the recombinant protein in previous studies. **Figure 8** shows that recombinant IFN- γ induces a significant increase in transcripts for the three chemokines analyzed. The increase in *cxcl10* was observed between 6 and 9 h, reaching a maximum of approximately 7070 times at 9 h of incubation. The increase in *cxcl11* expression is also statistically significant after 9 h of incubation (**Figure 8**). The increase is approximately 350,000 times and is achieved after 9 h of incubation, then transcript levels begin to decrease after 12 h of incubation (**Figure 8**). The kinetics of *cxcl9* upregulation are like those of *cxcl10* and *cxcl11*, although the increase does not reach statistical significance after treatment (**Figure 8**). Finally, cxcr3 expression shows an increasing trend; However, transcription levels do not show a significant difference with respect to time 0 h (**Figure 8**).

### 3.6 Viral stimuli regulate the expression of *cxcl9, cxcl10, cxcl11* and *cxcr3* in the SHK-1 cell line

**Figure 9:**
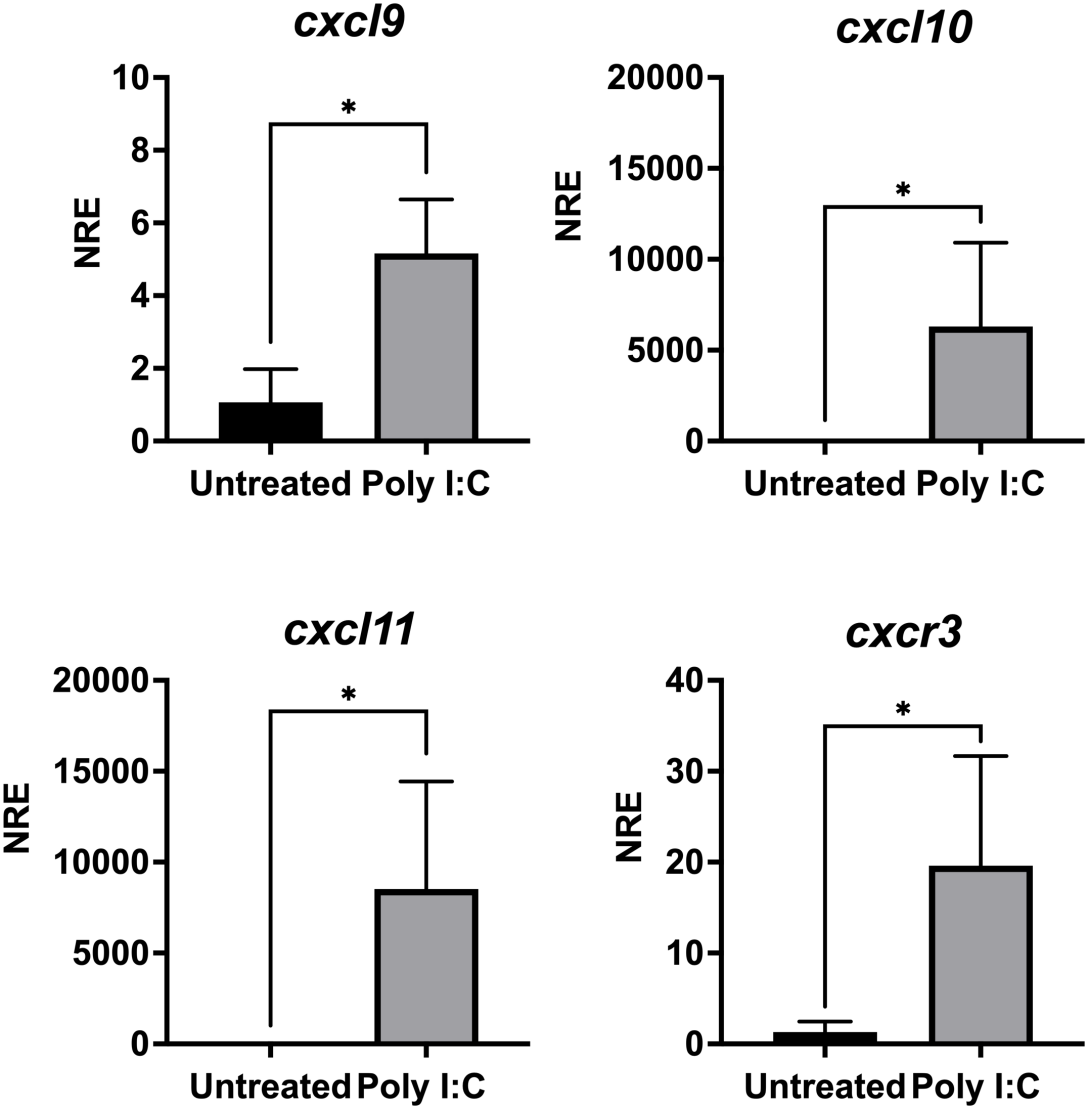
Poly I:C modulates the expression of genes *cxcl9, cxcl10, cxcl11* and *cxcr3* in SHK-1 cells. Cells were treated with poly I: C 24 h. Specific mRNA levels were measured by RT-qPCR and expressed gene expression was reported as relative to *β-Actin* expression (reference gene) and normalized with the relative expression of each gene in untreated cells (control group). The values obtained for each condition were expressed as normalized relative expression (NRE) ± standard deviation (SD) of 3 independent experiments (n=3). Differences between groups were determined by using a Mann-Whitney test. A p<0.05 was considered statistically significant. * p < 0.05.

**Figure 10:**
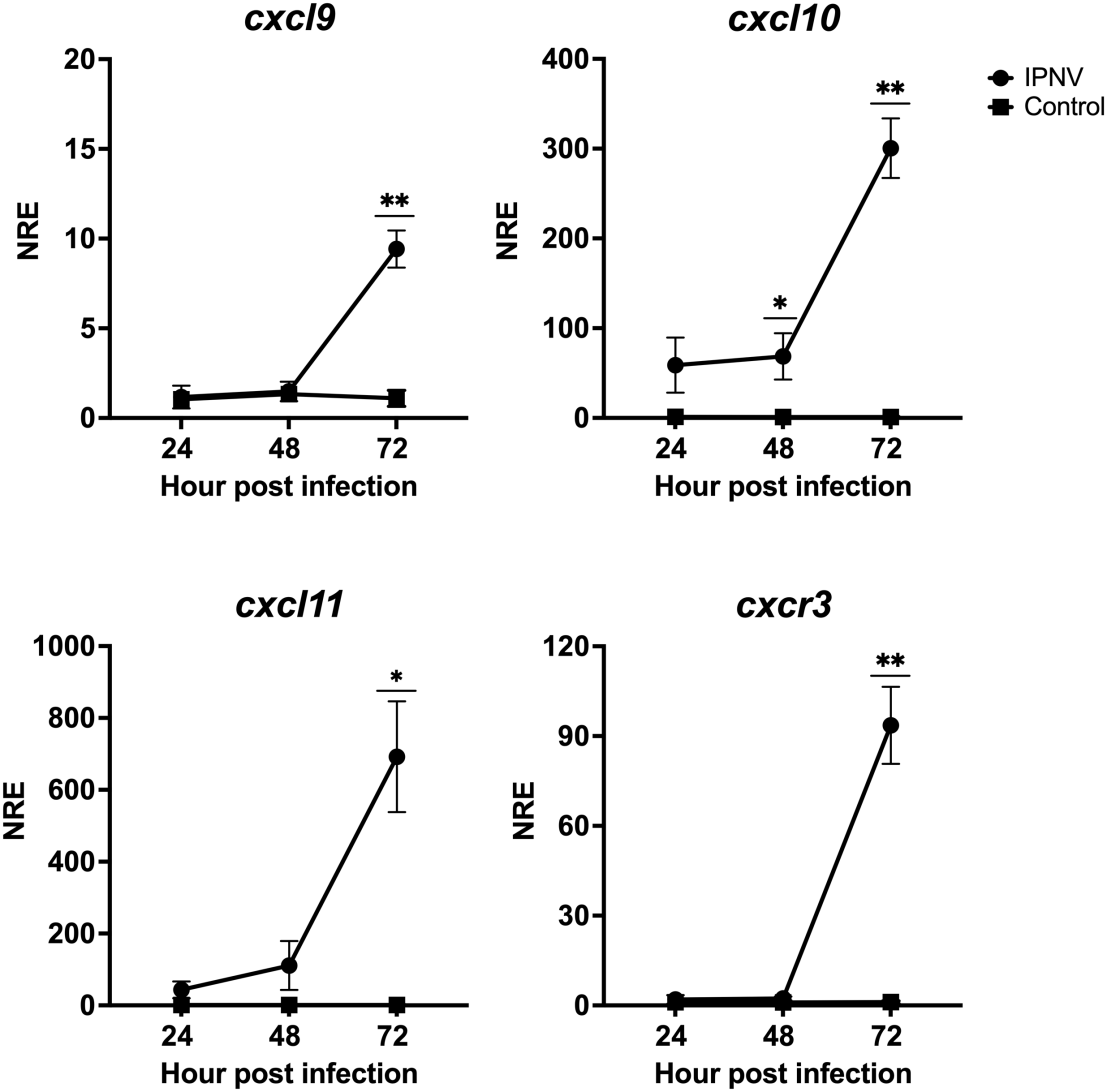
Infection with IPNV modulates the expression of genes *cxcl9, cxcl10, cxcl11* and *cxcr3* in CHSE-214 cells. Cells were infected with IPNV (MOI = 0,1). After 24, 48, and 72 hpi cells were collected, total RNA was isolated from CHSE-214 cells across all groups, and RT-qPCR was performed to quantify gene expression. Relative quantification of transcripts encoding CXCL9, CXCL10, CXCL11 and CXCR3 was conducted using the Pfaffl method. β-Actin expression (reference gene) and normalized with the relative expression of each gene in uninfected cells (control group). The values obtained for the control (square) and IPNV-infected (circles) condition were expressed as normalized relative expression (NRE) ± standard deviation (SD) of 3 independent experiments (n=3). Statistical analyses were performed using t-test with Welch’s correction. A p<0.05 was considered statistically significant. * p < 0.05; ** p < 0.01.

We next wondered whether poly I:C, a synthetic analog of double-stranded RNA (dsRNA) that mimics a viral molecular pattern, can also regulate the gene expression of the CXCL9-11/CXCR3 axis. SHK-1 cells were *in vitro* stimulated with poly I:C (10 μg/mL) or were kept without stimulation for 24 h to analyze further the transcriptional expression *cxcl9, cxl10, cxcl11*, and *cxcr3*. Results show that all genes are upregulated after poly I:C treatment (**Figure 9**). *cxcl9* expression increased approximately 5 times, *cxcl10* approximately 6,300 times, *cxcl11* increased 8,500 times, and *cxcr3* expression augmented approximately 19 times compared with the control group (**Figure 9**). On the other hand, the SHK-1 cells were infected with the Infectious Pancreatic Necrosis Virus at a multiplicity of infection (MOI) of 0.1, which is the dose that causes minimal cytopathic effect, for 24, 48, and 72 hours. The transcriptional expression of the three chemokines and the receptor only increased after 72 hours of infection compared to the levels of transcripts in uninfected cells (**Figure 10**). The expression levels increased approximately 9 times for *cxcl9*, 300 times for *cxcl-10*, 692 times for *cxcl11*, and 93 times for *cxcr3* (**Figure 10**), demonstrating that IPN viral infection can induce the expression of these chemokines and their receptor in Atlantic salmon probably to control cellular homing.

## 4 Discussion

The recruitment of leukocytes to injured tissues during infection is crucial for facilitating tissue repair and eliminating the underlying cause of inflammation (54). As a result, the role of chemokines, which guide this recruitment process, has been extensively investigated across various animal models (55). In this context, the current study aimed to investigate the genes encoding the CXCL9-11 chemokines and the CXCR3 receptor in Atlantic salmon. Our primary objective was to analyze the phylogenetic relationships of these proteins, their protein structure, and transcriptional expression regulation.

The analysis using a phylogenetic tree revealed CXCL9, CXCL10, and CXCL11 from fish, humans, and mice were group into three distinct clusters. Each cluster consisted mainly of one of the ligands but also included a small number of different annotated sequences. This supports the fact that the relationship between the CXCL9-11 genes found in fish remains unclear, as Chen pointed out in 2013 (10). In addition, this lack of consistency in naming these chemokines has led to varying nomenclature across different fish species. For example, Zebrafish exhibit a set of seven putative CXCL11 genes designated as CXCL11aa, ac, ad, ae, af, and ag genes (8). These genes are proposed to be named as CXCL11_L2 based on phylogenetic analysis by Chen (10). Furthermore, a CXCL10 gene previously identified in rainbow trout and other cyprinids, formerly called CXCb, has been categorized and recommended to be named CXCL11_L1 in a report by Torraca (56). The complexity of the phylogenetic analysis for the family of CXCL9-11 chemokines can be attributed to the increasing availability of genome sequences, which has led to the discovery of many more genes. Furthermore, the different human chemokines clustered together, which may be due to because mammalian chemokines originated from a relatively recent common ancestor (57). In contrast, the situation in fish species varied since they are very diverse organisms, and their chemokines evolve faster than other genes (6). Despite this, we have successfully established the orthologous relationships between the predicted sequences of the CXCL9-11 axis of Atlantic salmon and the genes from various fish species with diverse taxonomies, suggesting a strong possibility of functional relatedness.

The phylogenetic tree of two receptor families, the CXCR and CCR protein sequences, showed that the Atlantic salmon CXCR3 protein sequence was grouped with other teleost CXCR3 sequences having a common ancestor with the mouse and human genes, all of which indicate that they are orthologs. Results are consistent with the fact that genes of the CCR family cluster in a different clade. As expected, the Atlantic salmon sequences clustered with other salmon and trout species, indicating evolutionary conservation within the Salmonidae family. The phylogeny results also agree with previous studies (58) and turbot (59).

To get insights into the protein structure of the CXCL9-11 axis components, we first modeled the Atlantic salmon CXCL9, CXCL10 and CXCL11 chemokines. All of them exhibit the classic CXC chemokine structure characterized by a short N-terminal region, a large core stabilized by two disulfide bonds, three antiparallel beta-strands, and a C-terminal alpha-helix (60). All three showed high similarities with the main structural differences located in the loop, which is consistent with the fact that these loops shall be responsible for binding to different regions of the CXCR3 receptor as shown in mammals (61, 62). The core structure is well-ordered, but the N and C-terminals exhibit high conformational flexibility as has been reported for human chemokines (1, 61). The N-terminal region allows the chemokine to bind to the extracellular loops and transmembrane segments of the receptor, which is essential for signal transduction. Meanwhile, the C-terminal region can influence the overall conformation of the chemokine, affecting receptor interaction and the stability of the chemokine-receptor complex (63, 64). This flexibility allows chemokines to effectively bind to their receptors and other molecules, form gradients, and regulate the movement and activity of immune cells (64).

Regarding the CXCR3, the model revealed a conserved barrel-shaped protein structure with four highly preserved cysteines forming disulfide bonds involved in protein folding (65). The Atlantic salmon chemokine receptor sequences share only 38% identity with the human or mouse receptor. However, the three extracellular loops related to cytokine binding (66) and the intracellular loops containing the DRY motif involved in signal transduction (65) are all conserved in the salmon receptor. In Atlantic salmon, the predicted structure of CXCR3, obtained using the CXCR4 crystal, is highly conserved with the human CXCR3 receptor despite being modeled against bovine rhodopsin (61, 67).

Considering the ligand/receptor interactions (CXCL9-11/CXCR3), the best docking was selected using the HADDOCK score, which is a weighted sum of a variety of energy factors, including van der Waals, electrostatic, desolvation, and restraint violation energies (Evdw, Eelec, Edesol, and Eair, respectively) (52). All three complexes showed favorable binding energy, supporting the ligand-receptor interaction. The analysis indicated that the N-terminal of all three chemokines contributes to receptor binding affinity, which is consistent with prior findings (62), but only CXCL9 and CXCL11 show interactions between its loops and the CXCR3. Usually, research findings underscore the critical significance of the N-terminal regions in binding to CXCR3, primarily driven by van der Waals and electrostatic forces. CXCL9 showed the highest affinity for CXCR3 compared to CXCL11 and CXCL10, and CXCL11 has higher affinity than CXCL10. This appeared to be different in humans, as CXCL11 is the chemokine with the highest affinity for CXCR3, while CXCL10 shows a higher affinity than CXCL9 (62, 68, 69). The binding score of all these salmon complexes is related to the number of residues, and type of molecular interactions, such as the higher affinity of CXCL9 could be due to stabilization mediated by two ionic salt bridges and the lowest affinity ofCXCL10 due to the lack of interaction between its loop and the CXCR3. Altogether, this analysis of chemokine-receptor interactions provides valuable insights into the mechanisms of immune response in Atlantic salmon, but it is important to emphasize the need for further biochemical and biophysical methods to determine the ligand binding affinities between fish CXCR3 and its ligand chemokines, which has not been done for fish chemokines yet.

Regarding CXCL9-11/CXCR3 axis expression in Atlantic salmon, our analysis revealed that all four genes (*cxcl9, cxcl10, cxcl11*, and *cxcr3*) were expressed in both lymphoid and non-lymphoid tissues, indicating their potential involvement in various physiological processes. Few studies have reported the expression of *cxcl9* and *cxcl10* in Atlantic salmon tissues, i.e., transcripts have been detected in the gills and muscle of Atlantic salmon (20, 22, 70). No previous reports of *cxcl11* expression in Atlantic salmon tissues or cells exist. The CXCL9-11/CXCR3 transcripts have been reported in various other species of teleost fish (revised in Valdés et al., 2022 (12). For example, *cxcl9* is expressed in the preoptic nucleus, pituitary gland, and head kidney of carp (71), while CXCL10 is constitutively expressed in the gills, spleen, head kidney, and liver of rainbow trout (14), in the gills, thymus, mid-gut, spleen, liver, and kidney of *Salmo trutta* (23), and in the preoptic nucleus, pituitary gland, and head kidney of carp (71). Similarly, CXCL11 has been found in the muscle and spleen of rainbow trout (26, 27), and in embryos of *Danio rerio* (25, 56). CXCR3 receptor has also been identified in most tissues of rainbow trout (26, 27) and in ayu sweetfish (*Plecoglossus altivelis*) (29). Studies in carp showed that the recombinant CXCb protein (CXCL9-11 like) induced the chemotaxis of macrophages and granulocytes *in vitro*. Additionally, *in vitro*, CXCb also attracts cells from the lymphocyte/monocyte fraction (72). Overall, these chemokines seem to be found in most of the tissues tested across all the fish species studied. Their ubiquitous presence supports their role in recruiting leukocytes, which can occur in most tissues during both normal conditions and inflammation.

This study also showed that rIFN-γ increases the transcriptional levels of the chemokines CXCL9, CXCL10, and CXCL11, as well as the CXCR3 receptor in a macrophage-like cell line derived from a culture of head kidney leukocytes of Atlantic salmon (42), as occurs in carp where induction with recombinant carp IFN- γ stimulates induction of CXCb expression in carp phagocytes (73). The regulation of these chemokines and CXCR3 genes by IFN is consistent with what has been observed in mammals, where it helps the infiltration of effector T cells to sites of inflammation (74–76). Although the expression regulation of these genes by IFN-γ is a well-established fact in mammals, it has only been reported for *cxcl10* in rainbow trout RTS-11 cells (14), and for *cxcr3* in common carp (77). Since IFN-γ is a crucial cytokine produced as part of the antiviral response mechanisms, it is likely that the Atlantic salmon CXCL9-11/CXCR3 axis genes, induced by IFN-γ, also play a role in the immune response against virus infection. In this context, this study showed that poly I:C, a double-stranded RNA that mimics viral infection (78) increases the expression of all genes in the axis. A similar result has been reported in rainbow trout (14) and Nile tilapia (79), where stimulation with poly I:C induces the expression of *cxcl10*. Furthermore, it was observed that IPNV infection of SHK-1 cells also resulted in increased expression of all chemokines and CXCR3, which contrasts with other studies conducted in rainbow trout ovary after *in vivo* and *in vitro* infections (16); this discrepancy may be due to the specific organ, as no viral replication of IPNV has been detected in the ovary of rainbow trout (16). Additionally, in vertebrates, it has been observed that inflammatory responses triggered by pathogens in reproductive tissues are lower than in other tissues (80).

In our study, we observed that IPNV infection, as well as IFN-γ, leads to upregulation of CXCL9, CXCL10, CXCL11, and CXCR3 in a salmon immune cell line, suggesting that *in vivo*, these chemokines can help recruitment of immune cells, as part of the antiviral mechanisms of immunity in Atlantic salmon, just as it occurs in mammals. For example, in mammals, an increased accumulation of NK cells has been observed in the lungs of influenza A virus-infected mice, which depends on CXCR3 (81). Regarding the Respiratory syncytial virus, CXCL9 and CXCL10 are involved in T cell recruitment, and CXCL10 is also involved in the recruitment of dendritic cells. Therefore, in mammals, the chemokines of the CXCL9-11/CXCR3 axis play a role in immune cell recruitment during virus infections that are involved in virus clearance. However, in most cases, more studies are necessary to determine if the recruited cells (and, therefore, the chemokines) are beneficial or detrimental in the lungs (82).

In fishes, the role of CXCR3 in macrophage recruitment to the site of infection has been studied in zebrafish (83). To the best of our knowledge, no other functional studies to determine the role of the CXCL9-11/CXCR3 axis in leukocyte migration have been reported in fish species. In Atlantic salmon, we expect that a CXCL9-11/CXCR3-dependent recruitment of Th1 type cells, T CD8+ cells, and macrophages to the infection site should help eliminate virus-infected cells. This then remains to be demonstrated. In addition to the challenge of predicting whether other fish viruses would have a similar impact on the gene expression of the CXCL9-11/CXCR3 components, further research is needed to understand how the upregulation of CXCL9-11 and its receptor influences the course of viral infection in fish, including Atlantic salmon.

## Data Availability

The original contributions presented in the study are included in the article/**Supplementary Material**. Further inquiries can be directed to the corresponding author.

## Supplementary material

Supplementary Figure 1 rIFN-γ purification. Line 1: Whole protein extract from *E. coli BL21* transformed with the pET-15b-ssIFN-g1. Line 2: Whole protein extract from transformed *E. coli* treated with 1mM IPTG. Line 3: Molecular weight standard. Line 4: FPLC purified rIFN-γ.

Supplementary Figure 2 Evaluation of the expression of cxcl9, cxcl10, cxcl11, and cxcr3 following induction with LPS in SHK-1 cells. Cells were treated with 1 µg/mL of LPS for 9 h. Specific mRNA levels were measured by RT-qPCR and expressed gene expression was reported as relative to *β-Actin* expression (reference gene) and normalized with the relative expression of each gene in untreated cells (control group). The values obtained for each condition were expressed as normalized relative expression (NRE) ± standard deviation (SD) of 3 independent experiments (n=3). Differences between groups were determined with Kruskal-Wallis followed by Dunnet post hoc test.
