# Supplementary figures and images for: Expression and regulation of the CXCL9-11 chemokines and CXCR3 receptor in Atlantic salmon (Salmo salar)
Source: Front Immunol. 2024 Sep 5;15:1455457. doi: 10.3389/fimmu.2024.1455457 (PMC11410577; doi:10.3389/fimmu.2024.1455457)

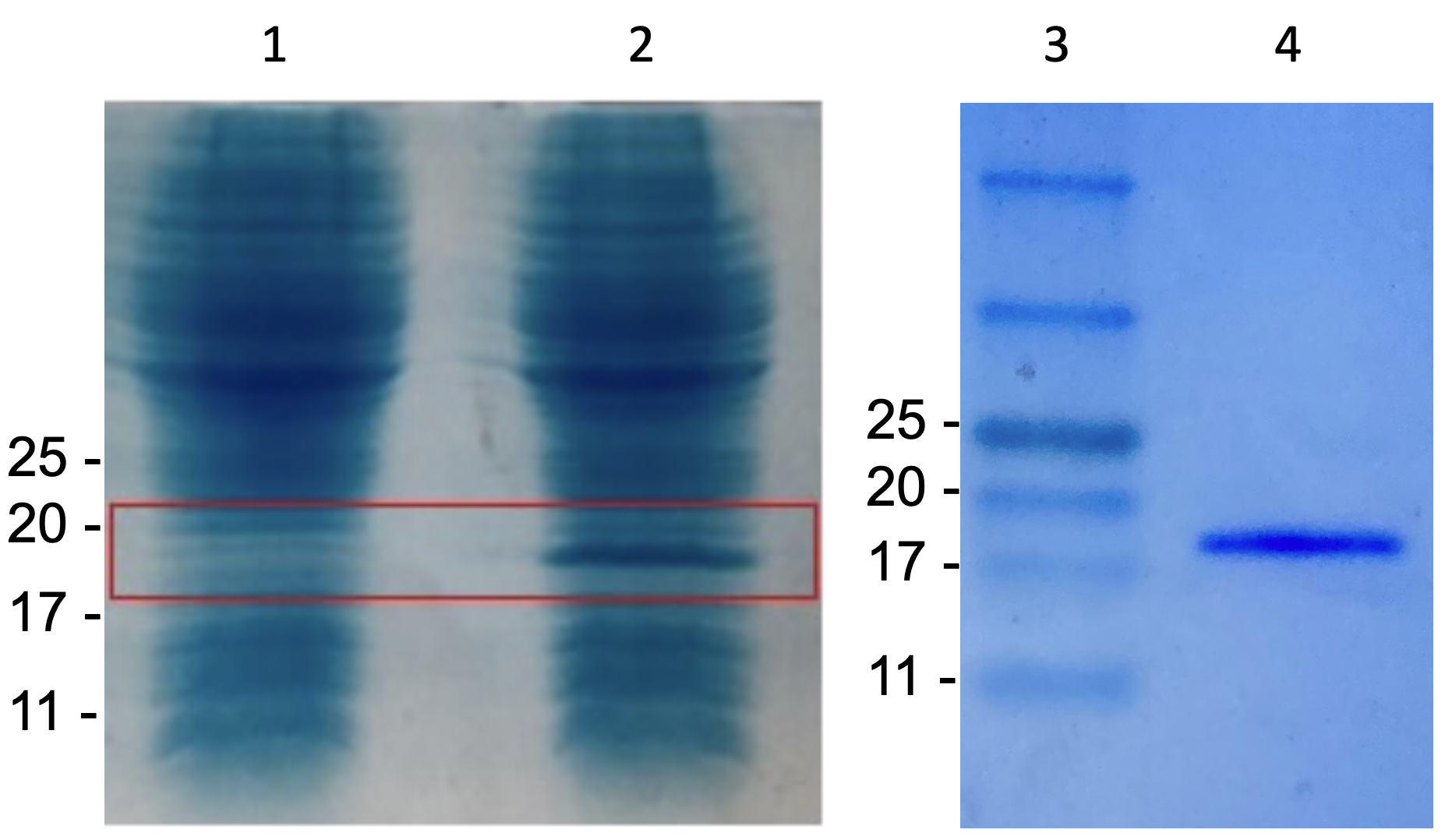

Supplement: Supplementary Figure 1 — rIFN-γ purification. Line 1: Whole protein extract from E. coli BL21 transformed with the pET-15b-ssIFN-g1. Line 2: Whole protein extract from transformed E. coli treated with 1mM IPTG. Line 3: Molecular weight standard. Line 4: FPLC purified rIFN-γ. [file Image1.tiff]

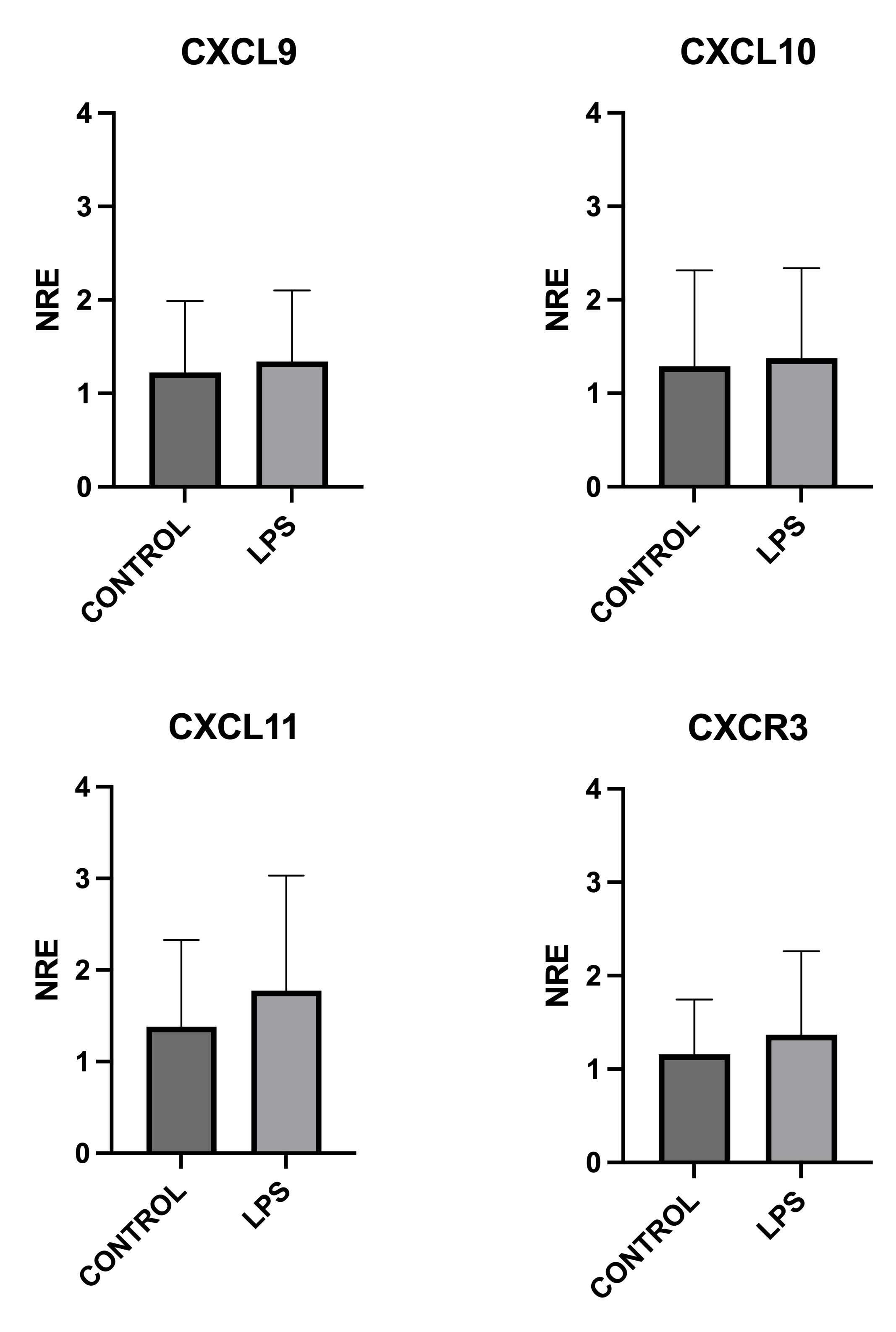

Supplement: Supplementary Figure 2 — Evaluation of the expression of cxcl9, cxcl10, cxcl11, and cxcr3 following induction with LPS in SHK-1 cells. Cells were treated with 1 µg/mL of LPS for 9 h. Specific mRNA levels were measured by RT-qPCR and expressed gene expression was reported as relative to β-Actin expression (reference gene) and normalized with the relative expression of each gene in untreated cells (control group). The values obtained for each condition were expressed as normalized relative expression (NRE) ± standard deviation (SD) of 3 independent experiments (n=3). Differences between groups were determined with Kruskal-Wallis followed by Dunnet post hoc test. [file Image2.tiff]
